# *KIT* Expression Is Regulated by DNA Methylation in Uveal Melanoma Tumors

**DOI:** 10.3390/ijms221910748

**Published:** 2021-10-04

**Authors:** Viera Horvathova Kajabova, Andrea Soltysova, Lucia Demkova, Paulina Plesnikova, Darina Lyskova, Alena Furdova, Pavel Babal, Bozena Smolkova

**Affiliations:** 1 Cancer Research Institute, Biomedical Research Center, Slovak Academy of Sciences, Dubravska Cesta 9, 845 05 Bratislava, Slovakia; viera.kajabova@savba.sk (V.H.K.); lucia.demkova@savba.sk (L.D.); 2 Department of Molecular Biology, Faculty of Natural Sciences, Comenius University in Bratislava, Ilkovicova 6, 841 04 Bratislava, Slovakia; andrea.soltysova@savba.sk; 3 Institute of Clinical and Translational Research, Biomedical Research Center, Slovak Academy of Sciences, Dubravska Cesta 9, 845 05 Bratislava, Slovakia; 4 Department of Ophthalmology, Faculty of Medicine, Comenius University in Bratislava, Ruzinovska 6, 821 01 Bratislava, Slovakia; plesnik.paula@gmail.com (P.P.); darina.lyskova@gmail.com (D.L.); alikafurdova@gmail.com (A.F.); 5 Department of Pathology, Faculty of Medicine, Comenius University in Bratislava, Sasinkova 4, 811 08 Bratislava, Slovakia; pavel.babal@fmed.uniba.sk

**Keywords:** uveal melanoma, *KIT*, MLPA, Monosomy 3, protein expression, DNA methylation

## Abstract

Uveal melanoma (UM) is an ocular tumor with a dismal prognosis. Despite the availability of precise molecular and cytogenetic techniques, clinicopathologic features with limited accuracy are widely used to predict metastatic potential. In 51 UM tissues, we assessed a correlation between the expression of nine proteins evaluated by immunohistochemistry (IHC) (Melan-A, S100, HMB45, Cyclin D1, Ki-67, p53, *KIT*, BCL2, and AIFM1) and the presence of UM-specific chromosomal rearrangements measured by multiplex ligation-dependent probe amplification (MLPA), to find IHC markers with increased prognostic information. Furthermore, mRNA expression and DNA methylation values were extracted from the whole-genome data, achieved by analyzing 22 fresh frozen UM tissues. *KIT* positivity was associated with monosomy 3, increasing the risk of poor prognosis more than 17-fold (95% CI 1.53–198.69, *p* = 0.021). A strong negative correlation was identified between mRNA expression and DNA methylation values for 12 of 20 analyzed positions, five located in regulatory regions of the *KIT* gene (r = −0.658, *p* = 0.001; r = −0.662, *p* = 0.001; r = −0.816; *p* < 0.001; r = −0.689, *p* = 0.001; r = −0.809, *p* < 0.001, respectively). DNA methylation β values were also inversely associated with *KIT* protein expression (*p* = 0.001; *p* = 0.001; *p* = 0.015; *p* = 0.025; *p* = 0.002). Our findings, showing epigenetic deregulation of *KIT* expression, may contribute to understanding the past failure to therapeutically target *KIT* in UM.

## 1. Introduction

Uveal melanoma (UM), a rare form of melanoma, is the most common intraocular cancer in adults [1]. It arises from melanocytes along the uveal tract, including the iris, ciliary body, and most often the choroid [2]. Almost half of UM patients develop metastases, which may be caused by a virtually undetectable neoplasm already present at the time of the primary tumor diagnosis [3]. UM spreads through the blood, with the liver being the preferred metastatic site, followed by the lungs and bones [4]. Due to the lack of effective therapies, outcomes for patients with metastatic disease remain dismal [5]. Risk of metastatic spread can be predicted through assessment of specific chromosome copy number alterations [6], gene expression profiles [7], and the mutation status of known UM driver genes [8].

The correlation between UM prognosis and particular chromosomal rearrangements was noted long ago [9]. The most frequent UM-specific aberrations include monosomy of chromosome 3 (M3), a gain in the short arm of chromosome 6 (6p), or a gain in the long arm of chromosome 8 (8q). The combination of M3 and polysomy 8q poses a high metastatic risk and presents a poor prognosis, similarly to the loss of the short arm of chromosome 8 (8p), the long arm of chromosome 6 (6q), and the short arm of chromosome 1 (1p) [10,11,12]. Conversely, the presence of 6p amplification represents a protective factor due to its association with a good prognosis and lowered metastatic risk [13]. Another way to predict the risk of metastasis is via gene expression analysis. A prospectively validated, commercially available 15-gene expression panel developed by Castle Biosciences categorizes patients as Class 1 (low metastatic risk) or Class 2 transcriptional subtype (high metastatic risk) [7,14]. Four molecular subsets were proposed recently, based on more complex classification [15,16]. Besides chromosomal rearrangements, this also includes generally mutually exclusive secondary driver mutations with prognostic potential, occurring in the *BAP1* (BRCA1-associated protein 1), *EIF1AX* (eukaryotic translation initiation factor 1A X-linked), or *SF3B1* (splicing factor 3b subunit 1) genes [17].

High expression of several immunohistochemical (IHC) markers in UM tumors, such as S100 (S100 calcium binding protein), Melan-A (Melanoma antigen recognized by T-cells 1), HMB45 (Human Melanoma Black), and some others, are regarded as clinically relevant diagnostic tools [18,19]. Cyclin D1 (Cyclin-D1-binding protein 1), Ki-67 (Proliferation marker protein Ki-67), and p53 (Cellular tumor antigen p53) positivities were linked with an unfavorable outcome [20]. Likewise, the overexpression of transmembrane tyrosine kinase receptor *KIT* (Mast/stem cell growth factor receptor Kit, alternative name CD117) was associated with poor prognosis in choroidal and ciliary body UM [21,22]. We recently identified 5-fold upregulation of its mRNA in M3 tumors compared to those with two copies of chromosome 3 (disomy 3, D3) [23]. In contrast to the normal ocular structures, UMs have also been characterized by upregulation of the anti-apoptotic protein BCL2 (Apoptosis regulator Bcl-2) [24]. Furthermore, we have shown that expression of AIFM1 (Apoptosis-inducing factor 1, mitochondrial), a protein with pro-apoptotic function in the nucleus and redox activity in mitochondria, was correlated with shorter survival in UM patients [25].

Epigenetic regulation plays the central role in time- and tissue-specific regulation of gene expression [26]. Epigenetic abnormalities contribute significantly to the development and progression of human malignancies. Deregulation of DNA methylation is one of the critical factors in resistance to current antitumoral therapies [27]. In close cooperation with histone modifications and noncoding RNA networks, DNA methylation controls normal cell development, chromatin stability, suppression of repetitive elements, and retrotransposition. Global hypomethylation occurs early in carcinogenesis, responsible for chromosomal instability and loss of imprinting [28]. Hypermethylation, associated with epigenetic silencing, is observed at multiple CpG sites in the regulatory regions of the genes, including CpG islands, shores, and shelves present inside or upstream of protein-coding genes promoters. Given the reversible nature of epigenetic changes, DNA methylation-based biomarkers have the potential to transform cancer diagnostics and treatment [29].

In this study, we assessed the association between the expression of nine proteins, Melan-A, S100, HMB45, Cyclin D1, Ki-67, p53, *KIT*, BCL2, AIFM1 and UM-specific chromosomal rearrangements in UM tissues, to increase prognostic accuracy of routinely investigated IHC markers. In addition, to better understand the molecular nature of their expression regulation, we also addressed DNA methylation in their regulatory and intragenic regions. A better understanding of epigenetic regulation might contribute to the development of effective therapy for poor prognosis UMs.

## 2. Results

### 2.1. Clinicopathologic Characteristics of the Patients

In the present study, 51 UM patients were enrolled between August 2018 and September 2020; nine (17.6%) had detectable metastases at the time of primary tumor diagnosis, one developed metastases eight months after surgery (Table 1). Only patients who underwent enucleation without prior treatment (70.6%) or enucleation after radiation therapy in the past (29.4%) were included. Their age ranged between 32 and 87 years, with a median of 65 years. The patient sex ratio was similar; male patients comprised 51.0%, female 49.0%. The right eyes were affected more often (56.9% right eye, 43.1% left eye). The majority of UMs (78.4%) arose from the choroid (C69.3), followed by the ciliary body (21.6%; C69.4). 51.0% of tumors were defined as spindle-cell, while 47.0% as epithelioid or mixed. A locally advanced disease, characterized by vascular cell invasion, was diagnosed in 17.6% cases, with lymphogenic invasion at 29.4%, perineural spread at 23.5%, and extrabulbar overgrowth detected in 15.7% of patients. No significant differences between studied clinicopathologic features in primary and metastatic patients were found except for extrabulbar overgrowth, more frequent in metastatic patients (40% vs. 9.8%) (Table 1).

### 2.2. Immunohistochemistry

**Figure 1:**
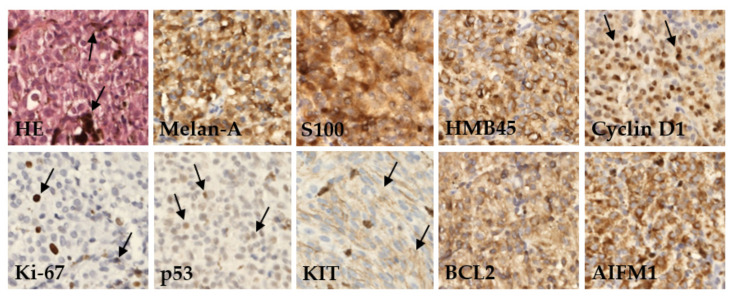
Immunohistochemical detection of various marker proteins’ expression in UM. In basic histological staining with hematoxylin and eosin (HE), the tumor cells showed a variable level of melanin content (arrows). In most cases, the tumors expressed melanoma markers like Melan-A, S100, and HMB45; variable amounts of prognostic markers as anti-apoptotic protein BCL2, Cyclin D1, tumor-suppressor protein p53, AIFM1, *KIT* protein, and proliferation rate protein Ki-67. Immunoperoxidase technique, diaminobenzidine, 200×.

We performed an IHC analysis of nine proteins in UM tissues assessed as part of a routine pathological examination (Figure 1).

Protein expression was categorized based on published cutoff values (see Section 4) into two categories, defined herein as positive or negative. The examined tumor tissues were characterized by a high frequency of positive Melan-A (96.0%), HMB45 (94.0%), and S100 (92.0%). Furthermore, a high expression level was found for AIFM1 (91.5%) and Cyclin D1 (84.0%) proteins. Conversely, the highest frequency of negative protein expression was detected for Ki-67 (74.0%) and p53 (70.0%). Differences in expression of these proteins between patients diagnosed with primary or metastatic disease were not significant (Table 2).

### 2.3. MLPA Analysis

**Figure 2:**
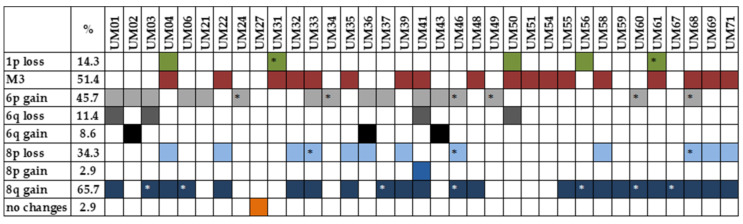
Presence of UM-specific chromosomal rearrangements detected by MLPA analysis. * partial loss/gain.

Multiplex ligation-dependent probe amplification (MLPA) analysis was used to identify chromosomal rearrangements in UM samples. Only tumor tissues not treated by stereotactic radiosurgery in the past were analyzed. M3 was detected in 18 tumors (51.4%). MLPA identified 1p loss/partial loss in five patients (14.3%), 6q loss in four patients (11.4%), and 6q gain in three (8.6%) patients; 6p gain/partial gain, associated with a good prognosis, was found in 16 patients (45.7%). Chromosomal abnormalities were also found on chromosome 8, with 8p loss/partial loss in 12 cases (34.3%), 8p gain in one case (2.9%), and 8q gain/partial gain in 23 (65.7%) cases. UM-specific chromosomal changes were not found in one patient (2.9%) (Figure 2). As M3 strongly correlates with metastatic death, while chromosome 8 gains occur later in UM tumorigenesis, for further analysis, we categorized patients based on M3 presence into two categories, M3 vs. D3.

The clinicopathologic features of patients and their association with M3 status are shown in Table 3.

All D3 patients were diagnosed with a malignant tumor of the choroid, while ciliary body tumors occurred in 38.9% of M3 patients (*p* = 0.004). Spindle-cell-type tumors were identified in 70.6% of D3, but only in 33.3% of M3 tumors, where epithelioid and mixed cell type prevailed (*p* = 0.025). This group’s high frequency of IIIA–C TNM stages (88.9% vs. 47.1% in D3, *p* = 0.008) might be associated with the nature of this study, where only patients treated by surgery were enrolled while low-stage patients were preferentially treated by radiotherapy.

Furthermore, the prognostic potential of selected IHC markers was also investigated (Table 4).

**Figure 3:**
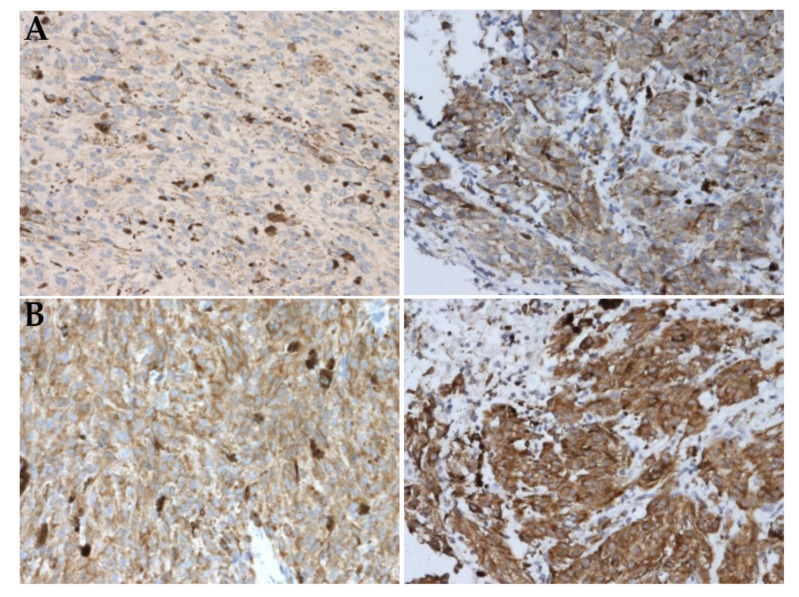
Immunohistochemical staining of *KIT* (**A**) and BCL2 (**B**) proteins in UM tumors. Negative/weak expression is shown on the left and positive on the right. Immunoperoxidase, diaminobenzidine, 200×.

Positive expression of two proteins, *KIT* and BCL2 was identified more frequently in poor-prognosis M3 tumors (72.2% vs. 18.8%, *p* = 0.002; 88.2% vs 50.0%, *p* = 0.017, respectively). Representative images of the tumors with negative/weak and positive expression of these two markers are shown in Figure 3.

The association between M3 and positive *KIT* expression was confirmed by a multivariate analysis (Table 5). *KIT* positivity increased the risk of unfavorable prognosis more than 17-fold (*p* = 0.021). The model successfully classified 75% of D3 and 100% of M3 patients, with an overall success rate of 88.2%. Among the other clinicopathologic parameters, epithelioid and mixed tumor types increased the risk of M3 18-fold (*p* = 0.029).

**Figure 4:**
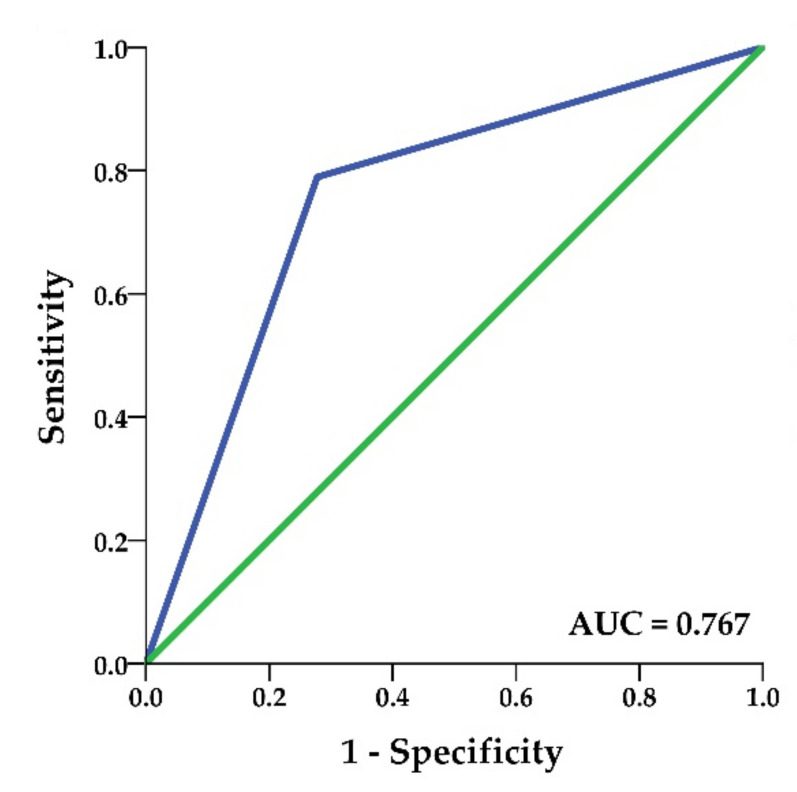
Receiver operator characteristic curve for *KIT* protein expression. AUC, area under the curve.

Receiver operating characteristic (ROC) curve analysis was performed to obtain diagnostic performance of *KIT* expression for M3. Area under the curve (AUC) was 0.767 (95% CI = 0.601–0.933; *p* = 0.008) (Figure 4). Using the 10% cutoff for *KIT* positivity, the sensitivity of this marker was 72%, while the specificity was 81%.

### 2.4. Gene Expression and DNA Methylation of *KIT* Gene

**Figure 5:**
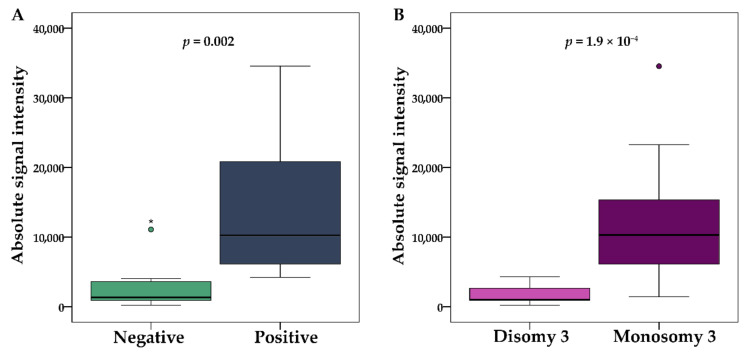
Differences in *KIT* mRNA absolute signal intensity between UM tumors with negative and positive protein expression (**A**) and D3 and M3 tumors (**B**). A horizontal line depicts the median. The boxes represent the interquartile range (IQR—values between the 75th and 25th percentiles). Values more than three IQRs are labeled as extremes (*), those between 1.5 IQRs and 3 IQRs as outliers (O).

Gene expression and DNA methylation data for *KIT* gene were extracted from the genome-wide transcriptomic and DNA methylation analysis, investigating 22 fresh frozen UM tumor samples from the same set (Supplementary Materials Table S1). Absolute signal intensity was used to compare gene expression and IHC data. Significantly higher *KIT* mRNA expression was found in tumors with positive protein expression (*p* = 0.002) (Figure 5A) and in M3 tumors (*p* = 1.9 × 10^−4^) (Figure 5B).

**Figure 6:**
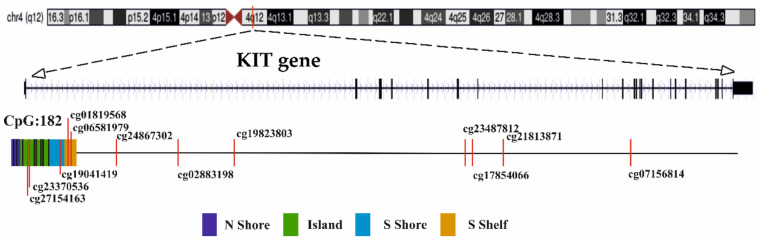
Location of studied CpG sites within *KIT* gene. Methylation β values in positions highlighted in red differed significantly between D3 and M3 tumors and between those with negative and positive protein expression.

The Infinium MethylationEPIC BeadChip array containing 20 probes for the *KIT* gene was used to analyze DNA methylation across different regions of the *KIT* gene. Probes were localized in N shore (*n* = 2), CpG island (*n* = 8), S shore (*n* = 1), S shelf (*n* = 2) and inside the gene (*n* = 7) (Figure 6). Individual methylation values (β value) were evaluated for each CpG site, ranging from 0 for unmethylated to 1 for fully methylated.

Methylation β values differed significantly in 12 positions between D3 and M3 tumors and between those with negative and positive protein expression (Table 6). In addition, a strong negative correlation between mRNA expression and DNA methylation; correlation coefficients (r) ranging between −0.425 and −0.809 were found in these positions.

**Figure 7:**
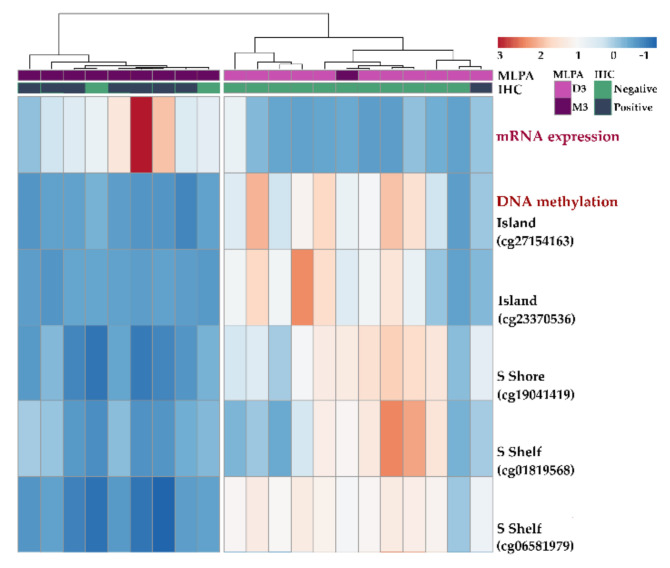
Heatmap showing the correlation between mRNA expression and DNA methylation. Only five representative probes located in regulatory regions of the *KIT* gene are shown. The red color in the histogram represents high signal intensity and methylation β value for individual positions, while the blue color represents a low signal intensity and methylation β value.

A heatmap was constructed based on normalized absolute signal intensity of mRNA expression and methylation β values for five CpGs located in regulatory regions (island, shores, shelves) (Figure 7). Samples were separated into two main clusters based on MLPA status and IHC categories.

**Figure 8:**
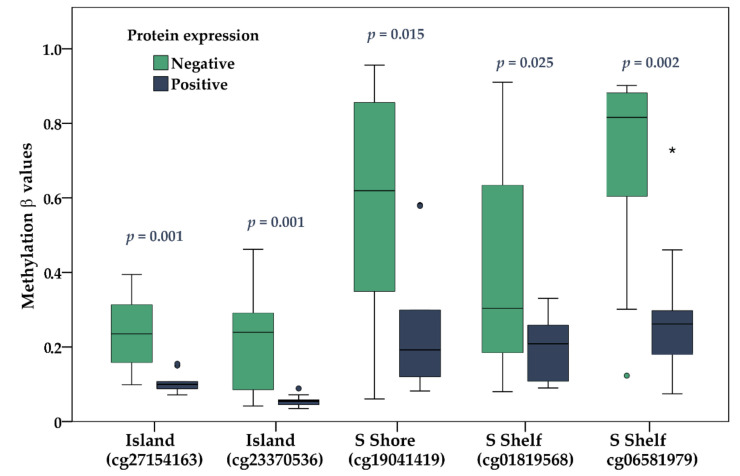
Methylation β values for five regulatory genomic regions of *KIT* negative and positive UM tumors. A horizontal line depicts the median. The boxes represent the interquartile range (IQR—values between the 75th and 25th percentiles). Values more than three IQRs are labeled as extremes (*), those between 1.5 IQRs and 3 IQRs as outliers (O).

Notably, DNA methylation of the *KIT* gene in studied genomic locations was inversely associated with protein expression (Figure 8).

## 3. Discussion

Accurate estimation of prognosis for patients with UM can be achieved by detecting genetic alterations or gene expression profiling in the tumor tissues. However, these opportunities are not always available, and clinicopathologic characteristics are frequently used in the clinic instead. Identifying novel markers with prognostic potential can thus help refine the prognosis of UM patients when the cytogenetic or molecular analysis is not accessible.

UM-specific chromosomal abnormalities are pretty common in UM tissues. Besides 1p loss, 3 loss, and 8q gain, which correlate with the metastatic risk, they also include 6p gain, associated with good prognosis [30]. Metastatic risk for patients with D3, disomy 6, and disomy 8 tumors was estimated to be 4%, increasing to 39% in those with M3 and 8q gain [31]. Chromosome 3 loss is associated with a 50% reduction in five-year survival [32]. Both M3 and 8q gain are related to other poor prognostic factors such as ciliary body involvement, epithelioid cells, large tumor basal diameter, high mitotic count, and closed connective tissue loops [33]. Given the high degree of correlation between M3 and Class 2 expression profile, M3 is a reliable prognostic marker [7,14,23].

MLPA, considered the gold standard method for the molecular analysis of copy number variations, was used to evaluate the association between M3 and protein expression of nine IHC markers, aiming to find those with increased prognostic information. *KIT* was the only protein independently associated with M3 in UM tumors after adjustment for clinical confounders. *KIT* is a transmembrane protein from the family of receptor tyrosine kinases, which, upon binding to its ligand SCF (stem cell factor), plays an essential role in regulating apoptosis, cell differentiation, proliferation, migration, and cell adhesion [34,35]. They are both essential for the proliferation and survival of normal melanocytes, with aberrant *KIT* signaling leading to melanoma [36]. One of *KIT* downstream targets is the melanocyte master regulator MITF (microphthalmia-associated transcription factor). Germline mutations in these three genes are associated with various pigmentation disorders [37].

Several studies explored the *KIT* expression and its prognostic significance in UM [21,22,38,39,40]. *KIT* positivity, detected in 20 of 48 (41.7%) samples analyzed in our study, agrees to 54% positivity reported by Lukenda et al., using the identical >10% cutoff [21]. However, with a 50% cutoff, Lüke et al. found high *KIT* expression in 63% of UM patients [39]. Different methodologies, antibody selection and specificity, arbitrary cutoff values, and antigen retrieval techniques can influence the interpretation of IHC analysis and thus be responsible for the inconsistent results reported to date [41].

Lukenda et al. demonstrated that *KIT*-positive patients (cutoff >10%, using 3-amino-9-ethylcarbazole chromogen) had a 4.13-fold higher risk (*p* = 0.017, 95% CI 1.289 to 13.223) of lethal outcome and shorter overall (*p* = 0.005) and disease-free survival (*p* = 0.009) in comparison with *KIT*-negative cases [21]. Accordingly, we found *KIT* among the top-ranked genes upregulated in M3 samples [23]. Recently, liquid biopsy proteomics data were published, revealing the biomarkers associated with metastatic risk. SCFR/c-Kit was among the top three proteins found to be upregulated in vitreous biopsies of UM patients compared to controls [42].

*KIT* overexpression was associated with cancer stem cell-like subpopulations, driving progression and therapeutic resistance in several cancer types [43,44]. To date, a substantial effort has been focused on targeting *KIT* by small molecule inhibitor imatinib, having dramatic activity against chemoresistant sarcoma and gastrointestinal stromal tumors, in which the clinical response correlates with the type of *KIT* mutations [45]. However, despite the relatively high *KIT* expression, zero response was found for metastatic UM in a clinical trial with imatinib [46]. Mutation analysis of UM tissues showed a lack of the mutations typical of gastrointestinal tumors, which led to the hypothesis that targeted therapies correlate with gene mutations and not with protein expression. It was later proven that *KIT* mutations occur in ocular melanomas at a frequency of 11%, with a limited correlation of KIT-positivity with mutational status [47]. Furthermore, in contrast with previous findings, the *KIT* protein was overexpressed in non-metastatic tumors, yet its expression was significantly reduced after metastasis.

Recently, epigenetic silencing of *KIT* gene by DNA methylation has been shown in cutaneous melanoma (CM) [36]. Surprisingly, the authors identified hypermethylation in 3/12 primary and 11/29 metastatic CMs. They concluded that loss of *KIT* by promoter hypermethylation suggests that distinct *KIT* signaling pathways play opposing roles in the pathogenesis of CM subtypes. DNA methylation-mediated loss of *KIT* expression during malignant transformation was also reported in breast cancer [48]. This methylation/expression paradox in mutation-free cancers was interpreted as a consequence of hypermethylation that might interfere with CTCF (CCCTC-binding factor) transcription repressor binding [49].

In our study, *KIT* overexpression was associated with poor prognosis of M3 UMs. Furthermore, a strong negative correlation was identified between *KIT* expression and DNA methylation. Evidence from epigenome-wide methylation studies suggests a higher correlation of DNA methylation with gene expression in shores than GpG islands [50]. DNA methylation within the gene body was not associated with gene expression changes consistently. In agreement with these reports, we found the strongest correlations between methylation β values and mRNA expression in S shore, S shelf, and gene body, rather than in CpG island. Interestingly, all studied CpGs located within the gene body were differentially methylated between M3 and D3 tumors and depending on the protein expression. Our data unambiguously support the role of DNA methylation in the regulation of *KIT* expression. However, given the differences between individual loci in terms of their potency in this regulatory function, the CpG region selection for association studies has to be done with care. Our findings can significantly impact the understanding of *KIT* overexpression in UM pathogenesis and lead to the discovery of new therapeutic approaches.

Several microRNAs (miRNAs), miR-193a, -193b, -221, -222, and -494, were demonstrated to repress *KIT* by directly targeting its mRNA. In addition, the miR-34 family was found to mediate *KIT* repression in a p53-dependent manner, reducing chemoresistance, migration, and stemness of cancer cells. Therefore, miR-34 mimics represent promising candidates for clinical applications [51]. Recently, enhancer domains of the *KIT* gene have been shown to be targetable by BET bromodomain inhibition in gastrointestinal stromal tumors, thus defining therapeutic vulnerability and the clinical strategy for targeting oncogenic kinases by the new generation of epigenetic drugs. The BET family of proteins regulates the chromatin state and transcription via binding acetylated proteins such as histones [52].

Furthermore, DNA methylation or histone modifications can be restored using nuclease-deficient Cas9 (dCas9) protein fused or noncovalently bound to epigenetic effectors. Epigenetic editing by CRISPR/dCas9 allows for locus-specific control of epigenetically regulated gene expression and provides a more specific alternative to epigenetic drugs [53]. Epigenetic therapy has taken a long time to be accepted for the treatment of solid tumors. Although more specific mechanisms need to be investigated, epigenome-targeted therapy seems to be a promising strategy for cancer treatment, including UM.

## 4. Materials and Methods

### 4.1. Patients

Between August 2018 and September 2020, 51 UM patients, treated by surgery (enucleation, exenteration) at the Department of Ophthalmology, Faculty of Medicine, Comenius University in Bratislava (University Hospital Bratislava in Slovakia), were enrolled. Among them, nine were diagnosed at stage IV, with metastases already present. One of the patients developed metastases eight months after primary UM treatment. Metastases were located in the liver (*n* = 3), lungs (*n* = 3), skin (*n* = 2), spine (*n* = 1), and pelvis (*n* = 1). The patient’s clinicopathologic characteristics are described in Table 1. The study was approved by the Ruzinov Hospital Bratislava Ethics Committee on December 12th, 2018 (number EK/250/2018). All subjects provided written informed consent. Only UM patients who underwent enucleation (*n* = 36) or enucleation after stereotactic radiosurgery in the past (*n* = 15) were included.

### 4.2. Immunohistochemistry

The formalin-fixed, paraffin-embedded tumors (FFPE) were stained with hematoxylin and eosin (HE) for routine histological examination and IHC stains at the Department of Pathology, Faculty of Medicine, Comenius University, Bratislava. Samples were evaluated by pathologists according to the WHO classification criteria [54]. The expression of nine proteins, Melan-A, S100, HMB45, Cyclin D1, Ki-67, p53, KIT, BCL2, and AIFM1, was evaluated by immunoperoxidase technique with the application of a FLEX EnVision Kit (Agilent Technologies, Santa Clara, CA, USA) in a DAKO Autostainer (Dako, Glostrup, Denmark).

Based on previously published cutoffs, protein expression was categorized into two groups, termed negative and positive (for cutoff values, see Table 2 and Table 4).

Positive expression for Melan-A, S100, and HMB45 antigens required ≥25% of melanoma cells displaying immunoreactivity [55]. Cyclin D1 and p53 expression were defined as positive when distinct nuclear staining was identified with >15% of positive tumor cells [20]. *KIT* expression was classified as positive if >10% of tumor cells displayed a distinct immunostaining pattern, irrespective of the staining intensity [21]. Cases with >15% positive nuclei were categorized as positive Ki-67 expression [56]. The slides stained for BCL2 were graded as negative (0), weakly positive (+), moderately positive (++), or strongly positive (+++) based on staining intensity rather than the number of cells stained [57]. Categories 0 and + were categorized as negative, while ++ and +++ as positive in our study. AIFM1 granular cytoplasmic positivity was evaluated as a quick score (QS) of staining intensity (negative, 0; weak, 1; moderate, 2; strong, 3) multiplied by density (<5%, 1; 6–20%, 2; 21–40%, 3; 41–60%, 4; 61–80%, 5; >81%, 6) [25].

### 4.3. MLPA Analysis

DNA from snap-frozen tumor tissues was isolated using a Gentra Puregene Tissue Kit (Qiagen, Hilden, Germany). Before extraction, tissue was mechanically disrupted on liquid nitrogen using a mortar and pestle. MLPA analysis was performed as described previously [23]. Briefly, a total of 100 ng of DNA extracted from frozen specimens was used to identify chromosomal aberrations in tumors by SALSA MLPA Probemix P027 Uveal melanoma (MRC Holland, Amsterdam, Netherlands). The kit contains probes located on chromosomes 1, 3, 6, and 8 (seven probes for 1p, 19 probes for chromosome 3, six probes for chromosome 6, and six probes for chromosome 8) and 12 control probes. Capillary electrophoresis was performed on a Genetic Analyzer 3130XL (Applied Biosystems, Foster City, CA, USA). Data were analyzed by Coffalyser software (MRC Holland, Amsterdam, Netherlands).

### 4.4. Microarray Analysis

According to the manufacturer’s instructions, RNA isolation was performed using approximately 34 mg of fresh frozen tumor tissue with the RNeasy Mini Kit (Qiagen, Venlo, Netherlands). An Agilent RNA 6000 Nano Kit was used for the analysis of the quality of isolated RNA. Only total RNA samples where RIN numbers were above 7.5 were used for microarray analysis, performed on 23 UM tissues as described previously [23]. Briefly, 100 ng of each sample was labeled using the Quick Amp Labeling kit (Agilent Technologies, Santa Clara, CA, USA), purified using the GeneJET^TM^ RNA Purification Kit (Thermo Fisher Scientific, Waltham, MA, USA), and subsequently hybridized using Gene Expression Hybridization Kit (Agilent Technologies, Santa Clara, CA, USA) onto SurePrint G3 Human Gene Expression 8 × 60K v2 Microarray Slide (Agilent Technologies, Santa Clara, CA, USA). After hybridization, slides were washed and scanned using a SureScan Microarray Scanner (Agilent Technologies, Santa Clara, CA, USA). Feature Extraction Software 12.0.3.2 (Agilent Technologies, Santa Clara, CA, USA) was used for TIFF image converting and processing, and gene expression analysis was performed in GeneSpring 14.9 GX software (Agilent Technologies, Santa Clara, CA, USA). The differences in gene expression were assessed by comparing analyzed conditions (e.g., M3 vs. D3 or protein expression) using a moderate T-test and fold change analysis (FC), considered significant when *p* < 0.05 and FC > 2.

### 4.5. Methylation Analysis

The Infinium MethylationEPIC BeadChip array (Illumina, Inc., San Diego, CA, USA) was used to assess the methylation status of the CpG sites. Across each array, M3 and D3 samples were randomly distributed. This high-throughput platform evaluated individual methylation levels (β values) for each CpG site, ranging from 0 for unmethylated to 1 for complete methylation. Data were analyzed using GenomeStudio v2011 (Illumina, Inc., San Diego, CA, USA). Significantly different methylations were obtained by using an unpaired Student’s *t*-test with *p* < 0.05 and the delta beta (Δβ) value ≥ 0.13.

### 4.6. Statistical Analysis

We used the following formula by Gass, TV = π/6 × (largest basal diameter × width × prominence), to calculate tumor volume [58]. Data were analyzed using IBM SPSS statistics software, version 23.0 software for Windows (IBM Corp., Armonk, NY, USA). Normality of distribution was tested by the Shapiro–Wilk test. Differences between studied groups were assessed using Pearson’s chi-square test, Fisher’s exact test for categorical variables, and the *t*-test or Mann–Whitney U test for continuous parameters. The Spearman correlation coefficient was calculated for correlation between non-normally distributed absolute signal intensity (mRNA) and methylation β values. The binary logistic regression was used to analyze the association between M3, clinicopathologic characteristics, and IHC markers. This determination included the computation of the risk estimates (odds ratio and 95% CI for the OR). The ROC analysis was applied to evaluate the diagnostic and predictive accuracy of *KIT* protein expression for M3, and AUC was computed as an effective measure of accuracy. All statistical tests were two-sided, and *p* < 0.05 was considered to be statistically significant.

## 5. Conclusions

The association found between *KIT* overexpression and poor prognosis M3 led to a new refinement of the prognostic significance of the *KIT* protein in UM. This can offer benefits for the clinical management of patients in situations where a molecular or cytogenetic analysis is not available. Learning more about *KIT* epigenetic regulation might contribute to a better understanding of past therapeutic failures and expand the treatment options for poor-prognosis UMs.

## Figures and Tables

**Table 1:** Clinicopathologic characteristics of the enrolled patients.

Clinicopathologic Characteristics	All *n* (%)	Primary UM *n* (%)	Metastatic UM *n* (%)	*p*-Value
Gender				
Male	26 (51.0)	23 (56.1)	3 (30.0)	0.139
Female	25 (49.0)	18 (43.9)	7 (70.0)	
Eye				
Right	29 (56.9)	25 (61.0)	4 (40.0)	0.230
Left	22 (43.1)	16 (39.0)	6 (60.0)	
Median age (range)	65.0 years (32–87)	66.5 years (35–80)	63.0 years (32–87)	0.658
Median tumor volume (range)	1.3 cm^3^ (0.3–2.6)	1.3 cm^3^ (0.3–2.6)	1.45 cm^3^ (0.3–2.6)	0.694
<1.55 cm^3^	34 (66.7)	27 (65.9)	7 (70.0)	0.803
≥1.55 cm^3^	17 (33.3)	14 (34.1)	3 (30.0)	
Diagnosis				
C69.3	40 (78.4)	34 (82.9)	6 (60.0)	0.114
C69.4	11 (21.6)	7 (17.1)	4 (40.0)	
Cell type *				
Spindle	26 (51.0)	22 (53.7)	4 (40.0)	0.612
Epitheloid and Mixed	24 (47.0)	18 (43.9)	6 (60.0)	
Therapy				
Enucleation	36 (70.6)	27 (65.9)	9 (90.0)	0.133
Enucleation after SRS ^1^	15 (29.4)	14 (34.1)	1 (10.0)	
Vascular invasion				
Present	9 (17.6)	6 (14.6)	3 (30.0)	0.253
Absent	42 (82.4)	35 (85.4)	7 (70.0)	
Lymphogenic invasion				
Present	15 (29.4)	10 (24.4)	5 (50.0)	0.111
Absent	36 (70.6)	31 (75.6)	5 (50.0)	
Perineural invasion				
Present	12 (23.5)	9 (22.0)	3 (30.0)	0.591
Absent	39 (76.5)	32 (78.0)	7 (70.0)	
Extrabulbar overgrowth				
Present	8 (15.7)	4 (9.8)	4 (40.0)	**0.018**
Absent	43 (84.3)	37 (90.2)	6 (60.0)	
TNM staging				
I–IIB	23 (45.1)	20 (48.8)	3 (30.0)	0.285
IIIA–C	28 (54.9)	21 (51.2)	7 (70.0)	

^1^ Stereotactic radiosurgery in the past; * cell type was not determined in one sample due to a high degree of cell dedifferentiation and necrosis.

**Table 2:** Protein expression of studied IHC markers.

IHC Markers	All *n* (%)	Primary UM *n* (%)	Metastatic UM *n* (%)	*p*-Value
Melan-A				
Positive, ≥25%	48 (96.0)	38 (95.0)	10 (100.0)	0.470
Negative	2 (4.0)	2 (5.0)	0 (0.0)	
S100				
Positive, ≥25%	46 (92.0)	37 (92.5)	9 (90.0)	0.794
Negative	4 (8.0)	3 (7.5)	1 (10.0)	
HMB45				
Positive, ≥25%	47 (94.0)	38 (95.0)	9 (90.0)	0.552
Negative	3 (6.0)	2 (5.0)	1 (10.0)	
Cyclin D1				
Positive, >15%	42 (84.0)	34 (85.0)	8 (80.0)	0.700
Negative	8 (16.0)	6 (15.0)	2 (20.0)	
Ki-67				
Positive, >15%	13 (26.0)	9 (22.5)	4 (40.0)	0.259
Negative	37 (74.0)	31 (77.5)	6 (60.0)	
p53				
Positive, >15%	15 (30.0)	12 (30.0)	3 (30.0)	1.000
Negative	35 (70.0)	28 (70.0)	7 (70.0)	
KIT				
Positive, >10%	20 (41.7)	15 (39.5)	5 (50)	0.548
Negative	28 (58.3)	23 (60.5)	5 (50)	
BCL2				
Positive, ≥ ++	32 (68.1)	25 (67.6)	7 (70.0)	0.884
Negative	15 (31.9)	12 (32.4)	3 (30.0)	
AIFM1				
Positive, QS ^1^ > 4	43 (91.5)	33 (89.2)	10 (100.0)	0.277
Negative	4 (8.5)	4 (10.8)	0 (0.0)	

^1^ Quick score.

**Table 3:** Association between diverse clinicopathologic characteristics and Monosomy 3 presence.

Clinicopathologic Characteristics	Disomy 3 *n* (%)	Monosomy 3 *n* (%)	*p*-Value
Gender ^1^			
Male	11 (64.7)	10 (55.6)	0.581
Female	6 (35.3)	8 (44.4)	
Eye			
Right	11 (64.7)	8 (44.4)	0.229
Left	6 (35.3)	10 (55.6)	
Median age (range)	69 years (35–87)	61 (32–81)	0.189
Median tumor volume (range)	1.4 cm^3^ (0.3–2.6)	1.5 cm^3^ (0.4–2.4)	0.681
<1.55 cm^3^	9 (52.9)	10 (55.6)	0.877
≥1.55 cm^3^	8 (47.1)	8 (44.4)	
Diagnosis			
C69.3	17 (100.0)	11 (61.1)	**0.004**
C69.4	0 (0.0)	7 (38.9)	
Cell type			
Spindle	12 (70.6)	6 (33.3)	**0.025**
Epitheloid and Mixed	5 (29.4)	12 (66.7)	
Vascular invasion			
Present	4 (23.5)	2 (11.1)	0.330
Absent	13 (76.5)	16 (88.9)	
Lymphogenic invasion			
Present	4 (23.5)	6 (33.3)	0.521
Absent	13 (76.5)	12 (66.7)	
Perineural invasion			
Present	5 (29.4)	5 (27.8)	0.915
Absent	12 (70.6)	13 (72.2)	
Extrabulbar overgrowth			
Present	1 (5.6)	5 (25.0)	0.101
Absent	17 (94.4)	15 (75.0)	
TNM staging			
I–IIB	9 (52.9)	2 (11.1)	**0.008**
IIIA–C	8 (47.1)	16 (88.9)	
Metastasis			
Present	3 (17.6)	6 (33.3)	0.289
Absent	14 (82.4)	12 (66.7)	

^1^ Only tumor tissues of the patients not treated by stereotactic radiosurgery in the past were analyzed (*n* = 36); of them, one sample was not available for MLPA analysis; therefore, only 35 samples were included in the analysis.

**Table 4:** Association between expression of studied proteins and Monosomy 3.

IHC Markers ^1^	Disomy 3 *n* (%)	Monosomy 3 *n* (%)	*p*-Value
Melan-A			
Positive, ≥25%	16 (94.1)	17 (94.4)	0.967
Negative	1 (5.9)	1 (5.6)	
S100			
Positive, ≥25%	16 (94.1)	15 (83.3)	0.316
Negative	1 (5.9)	3 (16.7)	
HMB45			
Positive, ≥25%	15 (88.2)	17 (94.4)	0.512
Negative	2 (11.8)	1 (5.6)	
Cyclin D1			
Positive, >15%	15 (88.2)	16 (88.9)	0.952
Negative	2 (11.8)	2 (11.1)	
Ki-67			
Positive, >15%	3 (17.6)	8 (44.4)	0.088
Negative	14 (82.4)	10 (55.6)	
p53			
Positive, >15%	5 (29.4)	7 (38.9)	0.555
Negative	12 (70.6)	11 (61.1)	
KIT			
Positive, >10%	3 (18.8)	13 (72.2)	**0.002**
Negative	13 (81.2)	5 (27.8)	
BCL2			
Positive, ≥ ++	8 (50.0)	15 (88.2)	**0.017**
Negative	8 (50.0)	2 (11.8)	
AIFM1			
Positive, QS ^2^ > 4	14 (93.3)	17 (100.0)	0.279
Negative	1 (6.7)	0 (0.0)	

^1^ Only tumor tissues of the patients not treated by stereotactic radiosurgery in the past were analyzed (*n* = 36); of them, one sample was not available for MLPA analysis; therefore, only 35 samples were included in the analysis; ^2^ Quick score.

**Table 5:** Binary logistic regression for the relationship between M3, clinicopathologic characteristics, and selected IHC markers (significance in univariate analysis *p* < 0.1).

Variable	OR	95% CI	*p*-Value
KIT positivity	17.41	1.53–198.69	**0.021**
Epithelioid and Mixed cell type	18.04	1.34–243.48	**0.029**
T stage IIIA–C	11.28	0.68–188.46	0.092
Constant			0.010

Variables entered in step 1: age, cell type, TNM staging, IHC markers with *p* value < 0.1 in univariate analysis (KIT, BCL2, Ki-67); −2 Log likelihood = 22.982; *R^2^* (Cox and Snell) = 0.485; *R^2^* (Nagelkerke) = 0.648.

**Table 6:** Association between *KIT* expression and DNA methylation evaluated for 20 distinct genomic positions of *KIT* gene.

Probe	UCSC Refgene Group	Feature	M3 vs. D3 *p*-Value ^£^	IHC * p-Value	Correlation Coefficient ^#^ *r* (*p*-Value)
cg16928454	TSS1500	N Shore	NS	NS	NS
cg06483432	TSS1500	N Shore	NS	NS	NS
cg26635759	TSS1500	Island	NS	NS	NS
**cg27154163**	TSS1500	Island	5.146 × 10^−4^	0.001	−0.658 (0.001)
**cg23370536**	TSS1500	Island	6.015 × 10^−4^	0.001	−0.662 (0.001)
cg11935854	5’UTR; 1st Exon	Island	NS	NS	NS
cg10087973	1st Exon	Island	NS	NS	NS
cg18836493	Body	Island	NS	NS	NS
cg17891820	Body	Island	NS	NS	NS
cg05786661	Body	Island	NS	NS	NS
**cg19041419**	Body	S Shore	3.866 × 10^−4^	0.015	−0.816 (<0.001)
**cg01819568**	Body	S Shelf	0.019	0.025	−0.689 (0.001)
**cg06581979**	Body	S Shelf	1.072 × 10^−6^	0.002	−0.809 (<0.000)
**cg24867302**	Body		0.015	0.011	−0.643 (0.002)
**cg02883198**	Body		0.005	0.028	−0.591 (0.005)
**cg19823803**	Body		0.006	0.044	−0.425 (0.055)
**cg23487812**	Body		1.685 × 10^−4^	0.002	−0.616 (0.003)
**cg17854066**	Body		2.281 × 10^−6^	0.001	−0.681 (0.001)
**cg21813871**	Body		2.908 × 10^−5^	0.001	−0.727 (<0.000)
**cg07156814**	Body		0.046	0.034	−0.532 (0.013)

**^£^** Difference between M3 and D3 gene expression data; * difference in methylation β values between *KIT* negative and positive tumors according to t-Test or Mann–Whitney U-test, respectively; **^#^** correlation between absolute signal intensity mRNA and methylation β values in individual positions. For each probe, the CpG feature and probe ID are listed; differentially methylated probes are in bold.

## Data Availability

All data supporting the reported results can be found as supplementary files.

## Supplementary Materials

The following are available online at https://www.mdpi.com/article/10.3390/ijms221910748/s1, Table S1: DNA methylation beta values for 20 Infinium MethylationEPIC BeadChip array probes targeting *KIT* gene.

## Abbreviations

AIFM1	Apoptosis-inducing factor 1: mitochondrial
AUC	Area under curve
BAP1	BRCA1 associated protein 1
BCL2	Apoptosis regulator Bcl-2
CI	Confidence interval
CM	Cutaneous melanoma
CpG	Cytosines followed by guanine residues
CTCF	Transcription repressor
Cyclin D1	Cyclin-D1-binding protein 1
D3	Disomy 3
EIF1AX	Eukaryotic translation initiation factor 1A X-linked
FC	Fold change analysis
FFPE	Formalin-fixed, paraffin-embedded tumors
HE	Hematoxylin and eosin
HMB45	Human melanoma black
IHC	Immunohistochemistry
IQR	Interquartile range
KIT	Mast/stem cell growth factor receptor Kit, alternative name CD117
Ki-67	Proliferation marker protein Ki-67
Melan A	Melanoma antigen recognized by T-cells 1
MITF	Melanocyte-inducing transcription factor
MLPA	Multiplex ligation-dependent probe amplification
M3	Monosomy of chromosome 3
OR	Odds ratio
p53	Cellular tumor antigen p53
r	Correlation coefficient
ROC	Receiver operating characteristic
SCF	Stem cell factor
SF3B1	Splicing factor 3b subunit 1
SRS	Stereotactic radiosurgery
S100	S100 calcium binding protein
TNM	Classification of malignant tumors, T—site and size of primary tumor, N—regional lymph nodes involvement, M—presence of distant metastasis
QS	Quick score
UM	Uveal melanoma
1p	Short arm of chromosome 1
6p	Short arm of chromosome 6
6q	Long arm of chromosome 6
8p	Short arm of chromosome 8
8q	Long arm of chromosome 8

## References

[1] Singh A.D., Turell M.E., Topham A.K. (2011). Uveal melanoma: Trends in incidence, treatment, and survival. Ophthalmology.

[2] McLaughlin C.C., Wu X.C., Jemal A., Martin H.J., Roche L.M., Chen V.W. (2005). Incidence of noncutaneous melanomas in the U.S. Cancer.

[3] Eskelin S., Pyrhönen S., Summanen P., Hahka-Kemppinen M., Kivelä T. (2000). Tumor doubling times in metastatic malignant melanoma of the uvea: Tumor progression before and after treatment. Ophthalmology.

[4] Diener-West M., Reynolds S.M., Agugliaro D.J., Caldwell R., Cumming K., Earle J.D., Hawkins B.S., Hayman J.A., Jaiyesimi I., Jampol L.M. (2005). Development of metastatic disease after enrollment in the COMS trials for treatment of choroidal melanoma: Collaborative Ocular Melanoma Study Group Report No. 26. Arch. Ophthalmol..

[5] Rantala E.S., Hernberg M., Kivelä T.T. (2019). Overall survival after treatment for metastatic uveal melanoma: A systematic review and meta-analysis. Melanoma Res..

[6] Damato B., Coupland S.E. (2009). Translating Uveal Melanoma Cytogenetics into Clinical Care. Arch. Ophthalmol..

[7] Onken M.D., Worley L.A., Ehlers J.P., Harbour J.W. (2004). Gene expression profiling in uveal melanoma reveals two molecular classes and predicts metastatic death. Cancer Res..

[8] Johansson P.A., Brooks K., Newell F., Palmer J.M., Wilmott J.S., Pritchard A.L., Broit N., Wood S., Carlino M.S., Leonard C. (2020). Whole genome landscapes of uveal melanoma show an ultraviolet radiation signature in iris tumours. Nat. Commun..

[9] Prescher G., Bornfeld N., Becher R. (1990). Nonrandom chromosomal abnormalities in primary uveal melanoma. J. Natl. Cancer Inst..

[10] Ehlers J.P., Worley L., Onken M.D., Harbour J.W. (2008). Integrative genomic analysis of aneuploidy in uveal melanoma. Clin. Cancer Res..

[11] Ewens K.G., Kanetsky P.A., Richards-Yutz J., Al-Dahmash S., De Luca M.C., Bianciotto C.G., Shields C.L., Ganguly A. (2013). Genomic Profile of 320 Uveal Melanoma Cases: Chromosome 8p-Loss and Metastatic Outcome. Investig. Ophthalmol. Vis. Sci..

[12] Versluis M., De Lange M.J., Van Pelt S.I., Ruivenkamp C.A., Kroes W.G., Cao J., Jager M.J., Luyten G.P., Van der Velden P.A. (2015). Digital PCR validates 8q dosage as prognostic tool in uveal melanoma. PLoS ONE.

[13] Amaro A., Gangemi R., Piaggio F., Angelini G., Barisione G., Ferrini S., Pfeffer U. (2017). The biology of uveal melanoma. Cancer Metastasis Rev..

[14] Onken M.D., Worley L.A., Char D.H., Augsburger J.J., Correa Z.M., Nudleman E., Aaberg T.M., Altaweel M.M., Bardenstein D.S., Finger P.T. (2012). Collaborative Ocular Oncology Group report number 1: Prospective validation of a multi-gene prognostic assay in uveal melanoma. Ophthalmology.

[15] Robertson A.G., Shih J., Yau C., Gibb E.A., Oba J., Mungall K.L., Hess J.M., Uzunangelov V., Walter V., Danilova L. (2017). Integrative Analysis Identifies Four Molecular and Clinical Subsets in Uveal Melanoma. Cancer Cell.

[16] Jager M.J., Shields C.L., Cebulla C.M., Abdel-Rahman M.H., Grossniklaus H.E., Stern M.H., Carvajal R.D., Belfort R.N., Jia R., Shields J.A. (2020). Uveal melanoma. Nat. Rev. Dis. Primers.

[17] Smit K.N., Jager M.J., De Klein A., Kiliç E. (2020). Uveal melanoma: Towards a molecular understanding. Prog. Retin. Eye Res..

[18] Schuster R., Bechrakis N.E., Stroux A., Busse A., Schmittel A., Scheibenbogen C., Thiel E., Foerster M.H., Keilholz U. (2007). Circulating tumor cells as prognostic factor for distant metastases and survival in patients with primary uveal melanoma. Clin. Cancer Res..

[19] Iwamoto S., Burrows R.C., Kalina R.E., George D., Boehm M., Bothwell M.A., Schmidt R. (2002). Immunophenotypic differences between uveal and cutaneous melanomas. Arch. Ophthalmol..

[20] Coupland S.E., Anastassiou G., Stang A., Schilling H., Anagnostopoulos I., Bornfeld N., Stein H. (2000). The prognostic value of cyclin D1, p53, and MDM2 protein expression in uveal melanoma. J. Pathol..

[21] Lukenda A., Dotlic S., Vukojevic N., Saric B., Vranic S., Zarkovic K. (2016). Expression and prognostic value of putative cancer stem cell markers CD117 and CD15 in choroidal and ciliary body melanoma. J. Clin. Pathol..

[22] All-Ericsson C., Girnita L., Müller-Brunotte A., Brodin B., Seregard S., Ostman A., Larsson O. (2004). c-Kit-dependent growth of uveal melanoma cells: A potential therapeutic target?. Investig. Ophthalmol. Vis. Sci..

[23] Soltysova A., Sedlackova T., Dvorska D., Jasek K., Chokhachi Baradaran P., Horvathova Kajabova V., Demkova L., Buocikova V., Kurucova T., Lyskova D. (2020). Monosomy 3 Influences Epithelial-Mesenchymal Transition Gene Expression in Uveal Melanoma Patients; Consequences for Liquid Biopsy. Int. J. Mol. Sci..

[24] Hussein M.R. (2005). Analysis of Bcl-2 protein expression in choroidal melanomas. J. Clin. Pathol..

[25] Krasnik V., Furdova A., Svetlosakova Z., Kobzova D., Gergisakova H., Feketeova L., Svetlosak M., Barta A., Babal P. (2017). Prognostic value of apoptosis inducing factor in uveal melanoma. Neoplasma.

[26] Zhang L., Lu Q., Chang C. (2020). Epigenetics in Health and Disease. Adv. Exp. Med. Biol..

[27] Romero-Garcia S., Prado-Garcia H., Carlos-Reyes A. (2020). Role of DNA Methylation in the Resistance to Therapy in Solid Tumors. Front. Oncol..

[28] Kim J., Bretz C.L., Lee S. (2015). Epigenetic instability of imprinted genes in human cancers. Nucleic Acids Res..

[29] Locke W.J., Guanzon D., Ma C., Liew Y.J., Duesing K.R., Fung K.Y.C., Ross J.P. (2019). DNA Methylation Cancer Biomarkers: Translation to the Clinic. Front. Genet..

[30] White V.A., Chambers J.D., Courtright P.D., Chang W.Y., Horsman D.E. (1998). Correlation of cytogenetic abnormalities with the outcome of patients with uveal melanoma. Cancer.

[31] Shields C.L., Say E.A.T., Hasanreisoglu M., Saktanasate J., Lawson B.M., Landy J.E., Badami A.U., Sivalingam M.D., Hauschild A.J., House R.J. (2017). Personalized Prognosis of Uveal Melanoma Based on Cytogenetic Profile in 1059 Patients over an 8-Year Period: The 2017 Harry S. Gradle Lecture. Ophthalmology.

[32] Prescher G., Bornfeld N., Hirche H., Horsthemke B., Jöckel K.H., Becher R. (1996). Prognostic implications of monosomy 3 in uveal melanoma. Lancet.

[33] Damato B., Dopierala J.A., Coupland S.E. (2010). Genotypic profiling of 452 choroidal melanomas with multiplex ligation-dependent probe amplification. Clin. Cancer Res..

[34] Lasota J., Miettinen M. (2008). Clinical significance of oncogenic *KIT* and PDGFRA mutations in gastrointestinal stromal tumours. Histopathology.

[35] Williams D.E., Eisenman J., Baird A., Rauch C., Van Ness K., March C.J., Park L.S., Martin U., Mochizuki D.Y., Boswell H.S. (1990). Identification of a ligand for the c-kit proto-oncogene. Cell.

[36] Dahl C., Abildgaard C., Riber-Hansen R., Steiniche T., Lade-Keller J., Guldberg P. (2015). KIT is a frequent target for epigenetic silencing in cutaneous melanoma. J. Investig. Dermatol..

[37] Lin J.Y., Fisher D.E. (2007). Melanocyte biology and skin pigmentation. Nature.

[38] Mouriaux F., Kherrouche Z., Maurage C.A., Demailly F.X., Labalette P., Saule S. (2003). Expression of the c-kit receptor in choroidal melanomas. Melanoma Res..

[39] Lüke J., Wegner J., Wegner R., Nassar K., Tatar O., Rohrbach J.M., Hilgers R.D., Lüke M., Grisanti S. (2011). Expression of c-Kit and its ligand SCF in primary uveal melanoma. Eur. J. Ophthalmol..

[40] Pereira P.R., Odashiro A.N., Marshall J.C., Correa Z.M., Belfort R., Burnier M.N. (2005). The role of c-kit and imatinib mesylate in uveal melanoma. J. Carcinog..

[41] De Wit N.J., Van Muijen G.N., Ruiter D.J. (2004). Immunohistochemistry in melanocytic proliferative lesions. Histopathology.

[42] Velez G., Nguyen H.V., Chemudupati T., Ludwig C.A., Toral M., Reddy S., Mruthyunjaya P., Mahajan V.B. (2021). Liquid biopsy proteomics of uveal melanoma reveals biomarkers associated with metastatic risk. Mol. Cancer.

[43] Harris K.S., Shi L., Foster B.M., Mobley M.E., Elliott P.L., Song C.J., Watabe K., Langefeld C.D., Kerr B.A. (2021). CD117/c-kit defines a prostate CSC-like subpopulation driving progression and TKI resistance. Sci. Rep..

[44] Foster B., Zaidi D., Young T., Mobley M., Kerr B. (2018). CD117/c-kit in cancer stem cell-mediated progression and therapeutic resistance. Biomedicines.

[45] Debiec-Rychter M., Dumez H., Judson I., Wasag B., Verweij J., Brown M., Dimitrijevic S., Sciot R., Stul M., Vranck H. (2004). Use of c-KIT/PDGFRA mutational analysis to predict the clinical response to imatinib in patients with advanced gastrointestinal stromal tumours entered on phase I and II studies of the EORTC Soft Tissue and Bone Sarcoma Group. Eur. J. Cancer.

[46] Hofmann U.B., Kauczok-Vetter C.S., Houben R., Becker J.C. (2009). Overexpression of the KIT/SCF in uveal melanoma does not translate into clinical efficacy of imatinib mesylate. Clin. Cancer Res..

[47] Wallander M.L., Layfield L.J., Emerson L.L., Mamalis N., Davis D., Tripp S.R., Holden J.A. (2011). KIT mutations in ocular melanoma: Frequency and anatomic distribution. Mod. Pathol..

[48] Janostiak R., Vyas M., Cicek A.F., Wajapeyee N., Harigopal M. (2018). Loss of c-KIT expression in breast cancer correlates with malignant transformation of breast epithelium and is mediated by *KIT* gene promoter DNA hypermethylation. Exp. Mol. Pathol..

[49] Chang S.-W., Chao W.-R., Ruan A., Wang P.-H., Lin J.-C., Han C.-P. (2015). A promising hypothesis of c-KIT methylation/ expression paradox in c-KIT (+) squamous cell carcinoma of uterine cervix ----- CTCF transcriptional repressor regulates c-KIT proto-oncogene expression. Diagn. Pathol..

[50] Martino D., Saffery R. (2015). Characteristics of DNA methylation and gene expression in regulatory features on the Infinium 450k Beadchip. bioRxiv.

[51] Siemens H., Jackstadt R., Kaller M., Hermeking H. (2013). Repression of c-Kit by p53 is mediated by miR-34 and is associated with reduced chemoresistance, migration and stemness. Oncotarget.

[52] Hemming M.L., Lawlor M.A., Andersen J.L., Hagan T., Chipashvili O., Scott T.G., Raut C.P., Sicinska E., Armstrong S.A., Demetri G.D. (2019). Enhancer Domains in Gastrointestinal Stromal Tumor Regulate *KIT* Expression and Are Targetable by BET Bromodomain Inhibition. Cancer Res..

[53] Chokhachi Baradaran P., Kozovska Z., Furdova A., Smolkova B. (2020). Targeting Epigenetic Modifications in Uveal Melanoma. Int. J. Mol. Sci..

[54] Edge S.B., Compton C.C. (2010). The American Joint Committee on Cancer: The 7th edition of the AJCC cancer staging manual and the future of TNM. Ann. Surg. Oncol..

[55] Viray H., Bradley W.R., Schalper K.A., Rimm D.L., Rothberg B.E.G. (2013). Marginal and joint distributions of S100, HMB-45, and Melan-A across a large series of cutaneous melanomas. Arch. Pathol. Lab. Med..

[56] Soliman N.A., Yussif S.M. (2016). Ki-67 as a prognostic marker according to breast cancer molecular subtype. Cancer Biol. Med..

[57] Chana J.S., Wilson G.D., Cree I.A., Alexander R.A., Myatt N., Neale M., Foss A.J., Hungerford J.L. (1999). c-myc, p53, and Bcl-2 expression and clinical outcome in uveal melanoma. Br. J. Ophthalmol..

[58] Gass J.D. (1985). Comparison of uveal melanoma growth rates with mitotic index and mortality. Arch. Ophthalmol..

